# Supplementary material for: Infectious Complications in Home Parenteral Nutrition: A Systematic Review and Meta-Analysis Comparing Peripherally-Inserted Central Catheters with Other Central Catheters
Source: Nutrients. 2019 Sep 4;11(9):2083. doi: 10.3390/nu11092083 (PMC6770172; doi:10.3390/nu11092083)
Supplement: Supplementary file 1 [file nutrients-11-02083-s001.pdf]

Supplemental data Table 1. The National Institutes of Health Quality Assessment Tool for Controlled Intervention Studies.

**Criteria Control Intervention Studies**	**Yes**	**No**	**Other (CD, NR, NA)***
1. Was the study described as randomized, a randomized trial, a randomized clinical trial, or an RCT?			
2. Was the method of randomization adequate (i.e., use of randomly generated assignment)?			
3. Was the treatment allocation concealed (so that assignments could not be predicted)?			
4. Were study participants and providers blinded to treatment group assignment?			
5. Were the people assessing the outcomes blinded to the participants' group assignments?			
6. Were the groups similar at baseline on important characteristics that could affect outcomes (e.g., demographics, risk factors, co-morbid conditions)?			
7. Was the overall drop-out rate from the study at endpoint 20% or lower of the number allocated to treatment?			
8. Was the differential drop-out rate (between treatment groups) at endpoint 15 percentage points or lower?			
9. Was there high adherence to the intervention protocols for each treatment group?			
10. Were other interventions avoided or similar in the groups (e.g., similar background treatments)?			
11. Were outcomes assessed using valid and reliable measures, implemented consistently across all study participants?			

Criteria Control Intervention Studies	Yes	No	Other (CD, NR, NA)*
12. Did the authors report that the sample size was sufficiently large to be able to detect a difference in the main outcome between groups with at least 80% power?			
13. Were outcomes reported or subgroups analyzed prespecified (i.e., identified before analyses were conducted)?			
14. Were all randomized participants analyzed in the group to which they were originally assigned, i.e., did they use an intention-to-treat analysis?			

Quality Rating (Good, Fair, or Poor)

Rater #1 initials:

Rater #2 initials:

Additional Comments (If POOR, please state why):

Quality Rating (Good, Fair, or Poor)

Rater #1 initials:

Rater #2 initials:

Additional Comments (If POOR, please state why):

*CD, cannot determine; NA, not applicable; NR, not reported

Guidance for Assessing the Quality of Controlled Intervention Studies

The guidance document below is organized by question number from the tool for quality assessment of controlled intervention studies.

Question 1. Described as randomized

Was the study described as randomized? A study does not satisfy quality criteria as randomized simply because the authors call it randomized; however, it is a first step in determining if a study is randomized

Questions 2 and 3. Treatment allocation–two interrelated pieces

Adequate randomization: Randomization is adequate if it occurred according to the play of chance (e.g., computer generated sequence in more recent studies, or random number table in older studies).
Inadequate randomization: Randomization is inadequate if there is a preset plan (e.g., alternation where every other subject is assigned to treatment arm or another method of allocation is used, such as time or day of hospital admission or clinic visit, ZIP Code, phone number, etc.). In fact, this is not randomization at all–it is another method of assignment to groups. If assignment is not by the play of chance, then the answer to this question is no.
There may be some tricky scenarios that will need to be read carefully and considered for the role of chance in assignment. For example, randomization may occur at the site level, where all individuals at a particular site are assigned to receive treatment or no treatment. This scenario is used for group-randomized trials, which can be truly randomized, but often are "quasi-experimental" studies with comparison groups rather than true control groups. (Few, if any, group-randomized trials are anticipated for this evidence review.)

Allocation concealment: This means that one does not know in advance, or cannot guess accurately, to what group the next person eligible for randomization will be assigned. Methods include sequentially numbered opaque sealed envelopes, numbered or coded containers, central randomization by a coordinating center, computer-generated randomization that is not revealed ahead of time, etc.

Questions 4 and 5. Blinding

Blinding means that one does not know to which group–intervention or control–the participant is assigned. It is also sometimes called "masking." The reviewer assessed whether each of the following was blinded to knowledge of treatment assignment: (1) the person assessing the primary outcome(s) for the study (e.g., taking the measurements such as blood pressure, examining health records for events such as myocardial infarction, reviewing and interpreting test results such as x ray or cardiac catheterization findings); (2) the person receiving the intervention (e.g., the patient or other study participant); and (3) the person providing the intervention (e.g., the physician, nurse, pharmacist, dietitian, or behavioral interventionist).

Generally placebo-controlled medication studies are blinded to patient, provider, and outcome assessors; behavioral, lifestyle, and surgical studies are examples of studies that are frequently blinded only to the outcome assessors because blinding of the persons providing and receiving the interventions is difficult in these situations. Sometimes the

individual providing the intervention is the same person performing the outcome assessment. This was noted when it occurred.

Question 6. Similarity of groups at baseline

This question relates to whether the intervention and control groups have similar baseline characteristics on average especially those characteristics that may affect the intervention or outcomes. The point of randomized trials is to create groups that are as similar as possible except for the intervention(s) being studied in order to compare the effects of the interventions between groups. When reviewers abstracted baseline characteristics, they noted when there was a significant difference between groups. Baseline characteristics for intervention groups are usually presented in a table in the article (often Table 1).

Groups can differ at baseline without raising red flags if: (1) the differences would not be expected to have any bearing on the interventions and outcomes; or (2) the differences are not statistically significant. When concerned about baseline difference in groups, reviewers recorded them in the comments section and considered them in their overall determination of the study quality.

Questions 7 and 8. Dropout

"Dropouts" in a clinical trial are individuals for whom there are no end point measurements, often because they dropped out of the study and were lost to followup.

Generally, an acceptable overall dropout rate is considered 20 percent or less of participants who were randomized or allocated into each group. An acceptable differential dropout rate is an absolute difference between groups of 15 percentage points at most (calculated by subtracting the dropout rate of one group minus the dropout rate of the other group). However, these are general rates. Lower overall dropout rates are expected in shorter studies, whereas higher overall dropout rates may be acceptable for studies of longer duration. For example, a 6-month study of weight loss interventions should be expected to have nearly 100 percent followup (almost no dropouts–nearly everybody gets their weight measured regardless of whether or not they actually received the intervention), whereas a 10-year study testing the effects of intensive blood pressure lowering on heart attacks may be acceptable if there is a 20-25 percent dropout rate, especially if the dropout rate between groups was similar. The panels for the NHLBI systematic reviews may set different levels of dropout caps.

Conversely, differential dropout rates are not flexible; there should be a 15 percent cap. If there is a differential dropout rate of 15 percent or higher between arms, then there is a serious potential for bias. This constitutes a fatal flaw, resulting in a poor quality rating for the study.

Question 9. Adherence

Did participants in each treatment group adhere to the protocols for assigned interventions? For example, if Group 1 was assigned to 10 mg/day of Drug A, did most

of them take 10 mg/day of Drug A? Another example is a study evaluating the difference between a 30-pound weight loss and a 10-pound weight loss on specific clinical outcomes (e.g., heart attacks), but the 30-pound weight loss group did not achieve its intended weight loss target (e.g., the group only lost 14 pounds on average). A third example is whether a large percentage of participants assigned to one group "crossed over" and got the intervention provided to the other group. A final example is when one group that was assigned to receive a particular drug at a particular dose had a large percentage of participants who did not end up taking the drug or the dose as designed in the protocol.

Question 10. Avoid other interventions

Changes that occur in the study outcomes being assessed should be attributable to the interventions being compared in the study. If study participants receive interventions that are not part of the study protocol and could affect the outcomes being assessed, and they receive these interventions differentially, then there is cause for concern because these interventions could bias results. The following scenario is another example of how bias can occur. In a study comparing two different dietary interventions on serum cholesterol, one group had a significantly higher percentage of participants taking statin drugs than the other group. In this situation, it would be impossible to know if a difference in outcome was due to the dietary intervention or the drugs.

Question 11. Outcome measures assessment

What tools or methods were used to measure the outcomes in the study? Were the tools and methods accurate and reliable–for example, have they been validated, or are they objective? This is important as it indicates the confidence you can have in the reported outcomes. Perhaps even more important is ascertaining that outcomes were assessed in the same manner within and between groups. One example of differing methods is self-report of dietary salt intake versus urine testing for sodium content (a more reliable and valid assessment method). Another example is using BP measurements taken by practitioners who use their usual methods versus using BP measurements done by individuals trained in a standard approach. Such an approach may include using the same instrument each time and taking an individual's BP multiple times. In each of these cases, the answer to this assessment question would be "no" for the former scenario and "yes" for the latter. In addition, a study in which an intervention group was seen more frequently than the control group, enabling more opportunities to report clinical events, would not be considered reliable and valid.

Question 12. Power calculation

Generally, a study's methods section will address the sample size needed to detect differences in primary outcomes. The current standard is at least 80 percent power to detect a clinically relevant difference in an outcome using a two-sided alpha of 0.05. Often, however, older studies will not report on power.

Question 13. Prespecified outcomes

Investigators should prespecify outcomes reported in a study for hypothesis testing– which is the reason for conducting an RCT. Without prespecified outcomes, the study may be reporting ad hoc analyses, simply looking for differences supporting desired findings. Investigators also should prespecify subgroups being examined. Most RCTs conduct numerous post hoc analyses as a way of exploring findings and generating additional hypotheses. The intent of this question is to give more weight to reports that are not simply exploratory in nature.

Question 14. Intention-to-treat analysis

Intention-to-treat (ITT) means everybody who was randomized is analyzed according to the original group to which they are assigned. This is an extremely important concept because conducting an ITT analysis preserves the whole reason for doing a randomized trial; that is, to compare groups that differ only in the intervention being tested. When the ITT philosophy is not followed, groups being compared may no longer be the same. In this situation, the study would likely be rated poor. However, if an investigator used another type of analysis that could be viewed as valid, this would be explained in the "other" box on the quality assessment form. Some researchers use a completers analysis (an analysis of only the participants who completed the intervention and the study), which introduces significant potential for bias. Characteristics of participants who do not complete the study are unlikely to be the same as those who do. The likely impact of participants withdrawing from a study treatment must be considered carefully. ITT analysis provides a more conservative (potentially less biased) estimate of effectiveness.

General Guidance for Determining the Overall Quality Rating of Controlled Intervention Studies

The questions on the assessment tool were designed to help reviewers focus on the key concepts for evaluating a study's internal validity. They are not intended to create a list that is simply tallied up to arrive at a summary judgment of quality.

Internal validity is the extent to which the results (effects) reported in a study can truly be attributed to the intervention being evaluated and not to flaws in the design or conduct of the study–in other words, the ability for the study to make causal conclusions about the effects of the intervention being tested. Such flaws can increase the risk of bias. Critical appraisal involves considering the risk of potential for allocation bias, measurement bias, or confounding (the mixture of exposures that one cannot tease out from each other). Examples of confounding include co-interventions, differences at baseline in patient characteristics, and other issues addressed in the questions above. High risk of bias translates to a rating of poor quality. Low risk of bias translates to a rating of good quality.

Fatal flaws: If a study has a "fatal flaw," then risk of bias is significant, and the study is of poor quality. Examples of fatal flaws in RCTs include high dropout rates, high differential dropout rates, no ITT analysis or other unsuitable statistical analysis (e.g., completers-only analysis).

Generally, when evaluating a study, one will not see a "fatal flaw;" however, one will find some risk of bias. During training, reviewers were instructed to look for the potential for bias in studies by focusing on the concepts underlying the questions in the tool. For any box checked "no," reviewers were told to ask: "What is the potential risk of bias that may be introduced by this flaw?" That is, does this factor cause one to doubt the results that were reported in the study?

NHLBI staff provided reviewers with background reading on critical appraisal, while emphasizing that the best approach to use is to think about the questions in the tool in determining the potential for bias in a study. The staff also emphasized that each study has specific nuances; therefore, reviewers should familiarize themselves with the key concepts.

Supplemental data Table 2. The National Institutes of Health Quality Assessment Tool for Observational Cohort and Cross-Sectional Studies.

**Criteria for Observational Cohort & Cross Sectional**	**Yes**	**No**	**Other (CD, NR, NA)***
1. Was the research question or objective in this paper clearly stated?			
2. Was the study population clearly specified and defined?			
3. Was the participation rate of eligible persons at least 50%?			
4. Were all the subjects selected or recruited from the same or similar populations (including the same time period)? Were inclusion and exclusion criteria for being in the study prespecified and applied uniformly to all participants?			
5. Was a sample size justification, power description, or variance and effect estimates provided?			
6. For the analyses in this paper, were the exposure(s) of interest measured prior to the outcome(s) being measured?			
7. Was the timeframe sufficient so that one could reasonably expect to see an association between exposure and outcome if it existed?			
8. For exposures that can vary in amount or level, did the study examine different levels of the exposure as related to the outcome (e.g., categories of exposure, or exposure measured as continuous variable)?			
9. Were the exposure measures (independent variables) clearly defined, valid, reliable, and implemented consistently across all study participants?			
10. Was the exposure(s) assessed more than once over time?			

**Criteria for Observational Cohort & Cross Sectional**	**Yes**	**No**	**Other (CD, NR, NA)***
11. Were the outcome measures (dependent variables) clearly defined, valid, reliable, and implemented consistently across all study participants?			
12. Were the outcome assessors blinded to the exposure status of participants?			
13. Was loss to follow-up after baseline 20% or less?			
14. Were key potential confounding variables measured and adjusted statistically for their impact on the relationship between exposure(s) and outcome(s)?			

Quality Rating (Good, Fair, or Poor)

Rater #1 initials:

Rater #2 initials:

Additional Comments (If POOR, please state why):

Quality Rating (Good, Fair, or Poor)

Rater #1 initials:

Rater #2 initials:

Additional Comments (If POOR, please state why):

*CD, cannot determine; NA, not applicable; NR, not reported

Guidance for Assessing the Quality of Observational Cohort and Cross-Sectional Studies

The guidance document below is organized by question number from the tool for quality assessment of observational cohort and cross-sectional studies.

Question 1. Research question

Did the authors describe their goal in conducting this research? Is it easy to understand what they were looking to find? This issue is important for any scientific paper of any type. Higher quality scientific research explicitly defines a research question.

Questions 2 and 3. Study population

Did the authors describe the group of people from which the study participants were selected or recruited, using demographics, location, and time period? If you were to conduct this study again, would you know who to recruit, from where, and from what time period? Is the cohort population free of the outcomes of interest at the time they were recruited?

An example would be men over 40 years old with type 2 diabetes who began seeking medical care at Phoenix Good Samaritan Hospital between January 1, 1990 and December 31, 1994. In this example, the population is clearly described as: (1) who (men over 40 years old with type 2 diabetes); (2) where (Phoenix Good Samaritan Hospital); and (3) when (between January 1, 1990 and December 31, 1994). Another example is women ages 34 to 59 years of age in 1980 who were in the nursing profession and had no known coronary disease, stroke, cancer, hypercholesterolemia, or diabetes, and were recruited from the 11 most populous States, with contact information obtained from State nursing boards.

In cohort studies, it is crucial that the population at baseline is free of the outcome of interest. For example, the nurses' population above would be an appropriate group in which to study incident coronary disease. This information is usually found either in descriptions of population recruitment, definitions of variables, or inclusion/exclusion criteria.

You may need to look at prior papers on methods in order to make the assessment for this question. Those papers are usually in the reference list.

If fewer than 50% of eligible persons participated in the study, then there is concern that the study population does not adequately represent the target population. This increases the risk of bias.

Question 4. Groups recruited from the same population and uniform eligibility criteria

Were the inclusion and exclusion criteria developed prior to recruitment or selection of the study population? Were the same underlying criteria used for all of the subjects involved? This issue is related to the description of the study population, above, and you may find the information for both of these questions in the same section of the paper.

Most cohort studies begin with the selection of the cohort; participants in this cohort are then measured or evaluated to determine their exposure status. However, some cohort studies may recruit or select exposed participants in a different time or place than unexposed participants, especially retrospective cohort studies–which is when data are obtained from the past (retrospectively), but the analysis examines exposures prior to outcomes. For example, one research question could be whether diabetic men with clinical depression are at higher risk for cardiovascular disease than those without clinical depression. So, diabetic men with depression might be selected from a mental health clinic, while diabetic men without depression might be selected from an internal medicine or endocrinology clinic. This study recruits groups from different clinic populations, so this example would get a "no."

However, the women nurses described in the question above were selected based on the same inclusion/exclusion criteria, so that example would get a "yes."

Question 5. Sample size justification

Did the authors present their reasons for selecting or recruiting the number of people included or analyzed? Do they note or discuss the statistical power of the study? This question is about whether or not the study had enough participants to detect an association if one truly existed.

A paragraph in the methods section of the article may explain the sample size needed to detect a hypothesized difference in outcomes. You may also find a discussion of power in the discussion section (such as the study had 85 percent power to detect a 20 percent increase in the rate of an outcome of interest, with a 2-sided alpha of 0.05). Sometimes estimates of variance and/or estimates of effect size are given, instead of sample size calculations. In any of these cases, the answer would be "yes."

However, observational cohort studies often do not report anything about power or sample sizes because the analyses are exploratory in nature. In this case, the answer would be "no." This is not a "fatal flaw." It just may indicate that attention was not paid to whether the study was sufficiently sized to answer a prespecified question–i.e., it may have been an exploratory, hypothesis-generating study.

Question 6. Exposure assessed prior to outcome measurement

This question is important because, in order to determine whether an exposure causes an outcome, the exposure must come before the outcome.

For some prospective cohort studies, the investigator enrolls the cohort and then determines the exposure status of various members of the cohort (large epidemiological

studies like Framingham used this approach). However, for other cohort studies, the cohort is selected based on its exposure status, as in the example above of depressed diabetic men (the exposure being depression). Other examples include a cohort identified by its exposure to fluoridated drinking water and then compared to a cohort living in an area without fluoridated water, or a cohort of military personnel exposed to combat in the Gulf War compared to a cohort of military personnel not deployed in a combat zone.

With either of these types of cohort studies, the cohort is followed forward in time (i.e., prospectively) to assess the outcomes that occurred in the exposed members compared to nonexposed members of the cohort. Therefore, you begin the study in the present by looking at groups that were exposed (or not) to some biological or behavioral factor, intervention, etc., and then you follow them forward in time to examine outcomes. If a cohort study is conducted properly, the answer to this question should be "yes," since the exposure status of members of the cohort was determined at the beginning of the study before the outcomes occurred.

For retrospective cohort studies, the same principal applies. The difference is that, rather than identifying a cohort in the present and following them forward in time, the investigators go back in time (i.e., retrospectively) and select a cohort based on their exposure status in the past and then follow them forward to assess the outcomes that occurred in the exposed and nonexposed cohort members. Because in retrospective cohort studies the exposure and outcomes may have already occurred (it depends on how long they follow the cohort), it is important to make sure that the exposure preceded the outcome.

Sometimes cross-sectional studies are conducted (or cross-sectional analyses of cohort-study data), where the exposures and outcomes are measured during the same timeframe. As a result, cross-sectional analyses provide weaker evidence than regular cohort studies regarding a potential causal relationship between exposures and outcomes. For cross-sectional analyses, the answer to Question 6 should be "no."

Question 7. Sufficient timeframe to see an effect

Did the study allow enough time for a sufficient number of outcomes to occur or be observed, or enough time for an exposure to have a biological effect on an outcome? In the examples given above, if clinical depression has a biological effect on increasing risk for CVD, such an effect may take years. In the other example, if higher dietary sodium increases BP, a short timeframe may be sufficient to assess its association with BP, but a longer timeframe would be needed to examine its association with heart attacks.

The issue of timeframe is important to enable meaningful analysis of the relationships between exposures and outcomes to be conducted. This often requires at least several years, especially when looking at health outcomes, but it depends on the research question and outcomes being examined.

Cross-sectional analyses allow no time to see an effect, since the exposures and outcomes are assessed at the same time, so those would get a "no" response.

Question 8. Different levels of the exposure of interest

If the exposure can be defined as a range (examples: drug dosage, amount of physical activity, amount of sodium consumed), were multiple categories of that exposure assessed? (for example, for drugs: not on the medication, on a low dose, medium dose, high dose; for dietary sodium, higher than average U.S. consumption, lower than recommended consumption, between the two). Sometimes discrete categories of exposure are not used, but instead exposures are measured as continuous variables (for example, mg/day of dietary sodium or BP values).

In any case, studying different levels of exposure (where possible) enables investigators to assess trends or dose-response relationships between exposures and outcomes–e.g., the higher the exposure, the greater the rate of the health outcome. The presence of trends or dose-response relationships lends credibility to the hypothesis of causality between exposure and outcome.

For some exposures, however, this question may not be applicable (e.g., the exposure may be a dichotomous variable like living in a rural setting versus an urban setting, or vaccinated/not vaccinated with a one-time vaccine). If there are only two possible exposures (yes/no), then this question should be given an "NA," and it should not count negatively towards the quality rating.

Question 9. Exposure measures and assessment

Were the exposure measures defined in detail? Were the tools or methods used to measure exposure accurate and reliable–for example, have they been validated or are they objective? This issue is important as it influences confidence in the reported exposures. When exposures are measured with less accuracy or validity, it is harder to see an association between exposure and outcome even if one exists. Also as important is whether the exposures were assessed in the same manner within groups and between groups; if not, bias may result.

For example, retrospective self-report of dietary salt intake is not as valid and reliable as prospectively using a standardized dietary log plus testing participants' urine for sodium content. Another example is measurement of BP, where there may be quite a difference between usual care, where clinicians measure BP however it is done in their practice setting (which can vary considerably), and use of trained BP assessors using standardized equipment (e.g., the same BP device which has been tested and calibrated) and a standardized protocol (e.g., patient is seated for 5 minutes with feet flat on the floor, BP is taken twice in each arm, and all four measurements are averaged). In each of these cases, the former would get a "no" and the latter a "yes."

Here is a final example that illustrates the point about why it is important to assess exposures consistently across all groups: If people with higher BP (exposed cohort) are seen by their providers more frequently than those without elevated BP (nonexposed group), it also increases the chances of detecting and documenting changes in health outcomes, including CVD-related events. Therefore, it may lead to the conclusion that

higher BP leads to more CVD events. This may be true, but it could also be due to the fact that the subjects with higher BP were seen more often; thus, more CVD-related events were detected and documented simply because they had more encounters with the health care system. Thus, it could bias the results and lead to an erroneous conclusion.

Question 10. Repeated exposure assessment

Was the exposure for each person measured more than once during the course of the study period? Multiple measurements with the same result increase our confidence that the exposure status was correctly classified. Also, multiple measurements enable investigators to look at changes in exposure over time, for example, people who ate high dietary sodium throughout the followup period, compared to those who started out high then reduced their intake, compared to those who ate low sodium throughout. Once again, this may not be applicable in all cases. In many older studies, exposure was measured only at baseline. However, multiple exposure measurements do result in a stronger study design.

Question 11. Outcome measures

Were the outcomes defined in detail? Were the tools or methods for measuring outcomes accurate and reliable–for example, have they been validated or are they objective? This issue is important because it influences confidence in the validity of study results. Also important is whether the outcomes were assessed in the same manner within groups and between groups.

An example of an outcome measure that is objective, accurate, and reliable is death–the outcome measured with more accuracy than any other. But even with a measure as objective as death, there can be differences in the accuracy and reliability of how death was assessed by the investigators. Did they base it on an autopsy report, death certificate, death registry, or report from a family member? Another example is a study of whether dietary fat intake is related to blood cholesterol level (cholesterol level being the outcome), and the cholesterol level is measured from fasting blood samples that are all sent to the same laboratory. These examples would get a "yes." An example of a "no" would be self-report by subjects that they had a heart attack, or self-report of how much they weigh (if body weight is the outcome of interest).

Similar to the example in Question 9, results may be biased if one group (e.g., people with high BP) is seen more frequently than another group (people with normal BP) because more frequent encounters with the health care system increases the chances of outcomes being detected and documented.

Question 12. Blinding of outcome assessors

Blinding means that outcome assessors did not know whether the participant was exposed or unexposed. It is also sometimes called "masking." The objective is to look for evidence in the article that the person(s) assessing the outcome(s) for the study (for example, examining medical records to determine the outcomes that occurred in the exposed and

comparison groups) is masked to the exposure status of the participant. Sometimes the person measuring the exposure is the same person conducting the outcome assessment. In this case, the outcome assessor would most likely not be blinded to exposure status because they also took measurements of exposures. If so, make a note of that in the comments section.

As you assess this criterion, think about whether it is likely that the person(s) doing the outcome assessment would know (or be able to figure out) the exposure status of the study participants. If the answer is no, then blinding is adequate. An example of adequate blinding of the outcome assessors is to create a separate committee, whose members were not involved in the care of the patient and had no information about the study participants' exposure status. The committee would then be provided with copies of participants' medical records, which had been stripped of any potential exposure information or personally identifiable information. The committee would then review the records for prespecified outcomes according to the study protocol. If blinding was not possible, which is sometimes the case, mark "NA" and explain the potential for bias.

Question 13. Followup rate

Higher overall followup rates are always better than lower followup rates, even though higher rates are expected in shorter studies, whereas lower overall followup rates are often seen in studies of longer duration. Usually, an acceptable overall followup rate is considered 80 percent or more of participants whose exposures were measured at baseline. However, this is just a general guideline. For example, a 6-month cohort study examining the relationship between dietary sodium intake and BP level may have over 90 percent followup, but a 20-year cohort study examining effects of sodium intake on stroke may have only a 65 percent followup rate.

Question 14. Statistical analyses

Were key potential confounding variables measured and adjusted for, such as by statistical adjustment for baseline differences? Logistic regression or other regression methods are often used to account for the influence of variables not of interest.

This is a key issue in cohort studies, because statistical analyses need to control for potential confounders, in contrast to an RCT, where the randomization process controls for potential confounders. All key factors that may be associated both with the exposure of interest and the outcome–that are not of interest to the research question–should be controlled for in the analyses.

For example, in a study of the relationship between cardiorespiratory fitness and CVD events (heart attacks and strokes), the study should control for age, BP, blood cholesterol, and body weight, because all of these factors are associated both with low fitness and with CVD events. Well-done cohort studies control for multiple potential confounders.

Some general guidance for determining the overall quality rating of observational cohort and cross-sectional studies

The questions on the form are designed to help you focus on the key concepts for evaluating the internal validity of a study. They are not intended to create a list that you simply tally up to arrive at a summary judgment of quality.

Internal validity for cohort studies is the extent to which the results reported in the study can truly be attributed to the exposure being evaluated and not to flaws in the design or conduct of the study–in other words, the ability of the study to draw associative conclusions about the effects of the exposures being studied on outcomes. Any such flaws can increase the risk of bias.

Critical appraisal involves considering the risk of potential for selection bias, information bias, measurement bias, or confounding (the mixture of exposures that one cannot tease out from each other). Examples of confounding include co-interventions, differences at baseline in patient characteristics, and other issues throughout the questions above. High risk of bias translates to a rating of poor quality. Low risk of bias translates to a rating of good quality. (Thus, the greater the risk of bias, the lower the quality rating of the study.)

In addition, the more attention in the study design to issues that can help determine whether there is a causal relationship between the exposure and outcome, the higher quality the study. These include exposures occurring prior to outcomes, evaluation of a dose-response gradient, accuracy of measurement of both exposure and outcome, sufficient timeframe to see an effect, and appropriate control for confounding–all concepts reflected in the tool.

Generally, when you evaluate a study, you will not see a "fatal flaw," but you will find some risk of bias. By focusing on the concepts underlying the questions in the quality assessment tool, you should ask yourself about the potential for bias in the study you are critically appraising. For any box where you check "no" you should ask, "What is the potential risk of bias resulting from this flaw in study design or execution?" That is, does this factor cause you to doubt the results that are reported in the study or doubt the ability of the study to accurately assess an association between exposure and outcome?

The best approach is to think about the questions in the tool and how each one tells you something about the potential for bias in a study. The more you familiarize yourself with the key concepts, the more comfortable you will be with critical appraisal. Examples of studies rated good, fair, and poor are useful, but each study must be assessed on its own based on the details that are reported and consideration of the concepts for minimizing bias.

Supplemental data text. References of the full-text articles screened-in but excluded for the meta-analysis, including reason for exclusion (Figure 1):

a. Inpatients, retrospective or pediatric patients.

1. Schmidt-Sommerfeld E, Snyder G, Rossi TM, Lebenthal E. Catheter-related complications in 35 children and adolescents with gastrointestinal disease on home parenteral nutrition. *JPEN J Parenter Enteral Nutr. 1990 Mar-Apr;14(2):148-51.*
2. DeLegge MH, Borak G, Moore N. “Central venous access in the home parenteral nutrition population-you PICC.” JPEN J Parenter Enteral Nutr. 2005 Nov-Dec;29(6):425-8.
3. Edakkanambeth-Varayil J, Whitaker JA, Okano A, et al. "Catheter Salvage After Catheter-Related Bloodstream Infection During Home Parenteral Nutrition" *JPEN J Parenter Enteral Nutr, 2017; Mar;41(3):481-488.*
4. Durkin MJ, Dukes JL, Reeds DN, et al. "A Descriptive Study of the Risk Factors Associated with Catheter-Related Bloodstream Infections in the Home Parenteral Nutrition Population" *JPEN J Parenter Enteral Nutr, 2016;Sep;40(7):1006-1013.*
5. Vashi, P. G., N. Virginkar, B. Popiel, P. Edwin, and D. Gupta. "Incidence of and Factors Associated with Catheter-Related Bloodstream Infection in Patients with Advanced Solid Tumors on Home Parenteral Nutrition Managed Using a Standardized Catheter Care Protocol." *BMC Infect Dis*, 2017; 17: 372.
6. Zhao, V. M., D. P. Griffith, H. M. Blumberg, N. J. Dave, C. H. Battey, T. A. McNally, K. A. Easley, J. R. Galloway, and T. R. Ziegler. "Characterization of Post-Hospital Infections in Adults Requiring Home Parenteral Nutrition." *Nutrition*, 2013; 29: 52-9.
7. Elfassy, S., Z. Kassam, F. Amin, K. J. Khan, S. Haider, and D. Armstrong. "Epidemiology and Risk Factors for Bloodstream Infections in a Home Parenteral Nutrition Program." *JPEN J Parenter Enteral Nutr*, 2015; 39: 147-53.
8. Christensen, L. D., M. Holst, L. F. Bech, L. Drustrup, L. Nygaard, A. Skallerup, H. H. Rasmussen, and L. Vinter-Jensen. "Comparison of Complications Associated with Peripherally Inserted Central Catheters and Hickman Catheters in Patients with Intestinal Failure Receiving Home Parenteral Nutrition. Six-Year Follow up Study." *Clin Nutr*, 2016; 35: 912-7.

b. No PICCs included.

1. Bond, A., A. Teubner, M. Taylor, C. Cawley, A. Abraham, M. Dibb, P. R. Chadwick, M. Soop, G. Carlson, and S. Lal. "Assessing the Impact of Quality Improvement Measures on Catheter Related Blood Stream Infections and Catheter Salvage: Experience from a National Intestinal Failure Unit." *Clin Nutr* 37, no. 6 Pt A (2018): 2097-101.
2. Tribler, Siri, Christopher F. Brandt, Mark Hvistendahl, Michael Staun, Per Brøbech, Claus E. Moser, and Palle B. Jeppesen. "Catheter-Related Bloodstream Infections in Adults Receiving Home Parenteral Nutrition." *Journal of Parenteral and Enteral Nutrition* (2017).
3. Tribler, S., C. F. Brandt, K. A. Fuglsang, M. Staun, P. Broebech, C. E. Moser, T. Scheike, and P. B. Jeppesen. "Catheter-Related Bloodstream Infections in Patients with Intestinal Failure Receiving Home Parenteral Support: Risks Related to a Catheter-Salvage Strategy." *Am J Clin Nutr* 107, no. 5 (2018): 743-53.
4. Guglielmi, F. W., N. Regano, S. Mazzuoli, M. Rizzi, S. Fregnan, G. Leogrande, I. Addante, and A. Guglielmi. "Catheter-Related Complications in Long-Term Home Parenteral Nutrition Patients with Chronic Intestinal Failure." *J Vasc Access* 13, no. 4 (2012): 490-7.
5. Dibb, M. J., A. Abraham, P. R. Chadwick, J. L. Shaffer, A. Teubner, G. L. Carlson, and S. Lal. "Central Venous Catheter Salvage in Home Parenteral Nutrition Catheter-Related Bloodstream Infections: Long-Term Safety and Efficacy Data." *JPEN J Parenter Enteral Nutr* 40, no. 5 (2016): 699-704.
6. Lawinski, M., K. Majewska, L. Gradowski, I. Foltyn, and P. Singer. "A Comparison of Two Methods of Treatment for Catheter-Related Bloodstream Infections in Patients on Home Parenteral Nutrition." *Clin Nutr* 34, no. 5 (2015): 918-22.
7. Lawinski, M., K. Forysinski, A. Bzikowska, J. Z. Kostro, A. Gradowska, and M. Pertkiewicz. "A Comparison of Two Methods of Treatment for Central Catheter Tunnel Phlegmon in Home Parenteral Nutrition Patients." *Prz Gastroenterol* 11, no. 3 (2016): 170-75.
8. Lawinski, M., K. Majewska, I. Foltyn, and A. Gradowska. "The Efficacy of Alcohol-Antibiotic Lock Therapy for Treatment of Catheter Related Bloodstream Infections in Patients Receiving Home Parenteral Nutrition." *Pol Przegl Chir* 86, no. 12 (2015): 563-8.
9. Nielsen, X. C., M. Chen, A. M. Hellesoe, P. B. Jeppesen, J. Gyldenlykke, M. Tvede, and L. P. Andersen. "Etiology and Epidemiology of Catheter Related Bloodstream Infections in Patients

Receiving Home Parenteral Nutrition in a Gastromedical Center at a Tertiary Hospital in Denmark." *Open Microbiol J* 6 (2012): 98-101.

10. Beraud, G., D. Seguy, S. Alfandari, X. Lenne, F. Leburgue, K. Faure, and B. Guery. "Factors Associated with Recurrence of Catheter-Related Bloodstream Infections in Home Parenteral Nutrition Patients." *Eur J Clin Microbiol Infect Dis* 31, no. 11 (2012): 2929-33.
11. Burden, S., M. Hemstock, M. Taylor, A. Teubner, N. Roskell, A. MacCulloch, A. Abraham, and S. Lal. "The Impact of Home Parenteral Nutrition on the Burden of Disease Including Morbidity, Mortality and Rate of Hospitalisations." *Clin Nutr ESPEN* 28 (2018): 222-27.
12. Harrison, E., A. L. Herrick, M. Dibb, J. T. McLaughlin, and S. Lal. "Long-Term Outcome of Patients with Systemic Sclerosis Requiring Home Parenteral Nutrition." *Clin Nutr* 34, no. 5 (2015): 991-6.
13. Pichitchaipitak, O., S. Ckumdee, S. Apivanich, D. Chotiprasitsakul, and P. C. Shantavasinkul. "Predictive Factors of Catheter-Related Bloodstream Infection in Patients Receiving Home Parenteral Nutrition." *Nutrition* 46 (2018): 1-6.
14. Salonen, B. R., S. L. Bonnes, N. Vallumsetla, J. E. Varayil, M. S. Mundi, and R. T. Hurt. "A Prospective Double Blind Randomized Controlled Study on the Use of Ethanol Locks in Hpn Patients." *Clin Nutr* 37, no. 4 (2018): 1181-85.
15. Buchman, A. L., M. Opilla, M. Kwasny, T. G. Diamantidis, and R. Okamoto. "Risk Factors for the Development of Catheter-Related Bloodstream Infections in Patients Receiving Home Parenteral Nutrition." *JPEN J Parenter Enteral Nutr* 38, no. 6 (2014): 744-9.
16. Klek, S., K. Szczepanek, A. Hermanowicz, and A. Galas. "Taurolidine Lock in Home Parenteral Nutrition in Adults: Results from an Open-Label Randomized Controlled Clinical Trial." *JPEN J Parenter Enteral Nutr* 39, no. 3 (2015): 331-5.
17. Olthof, E. D., M. W. Versleijen, G. Huisman-de Waal, T. Feuth, W. Kievit, and G. J. Wanten. "Taurolidine Lock Is Superior to Heparin Lock in the Prevention of Catheter Related Bloodstream Infections and Occlusions." *PLoS One* 9, no. 11 (2014): e111216.
18. Tribler, S., C. F. Brandt, A. H. Petersen, J. H. Petersen, K. A. Fuglsang, M. Staun, P. Broebech, C. E. Moser, and P. B. Jeppesen. "Taurolidine-Citrate-Heparin Lock Reduces Catheter-Related Bloodstream Infections in Intestinal Failure Patients Dependent on Home Parenteral Support: A Randomized, Placebo-Controlled Trial." *Am J Clin Nutr* 106, no. 3 (2017): 839-48

19. Touré A, Lauverjat M, Peraldi C, Boncompain-Gerard M, Gelas P, Barnoud D, Chambrier C. "Taurolidine lock solution in the secondary prevention of central venous catheter-associated bloodstream infection in home parenteral nutrition patients." *Clin Nutr. 2012 Aug;31(4):567-70. doi: 10.1016/j.clnu.2012.01.001. Epub 2012 Jan 28.*

20. Lee AM, Gabe SM, Nightingale JM, Burke M. "Oral health, dental prophylaxis and catheter related bloodstream infections in home parenteral nutrition patients: results of a UK survey and cohort study." *Br Dent J. 2012 Jan 27;212(2):E4. doi: 10.1038/sj.bdj.2012.50.*

21. Santarpia L, Alfonsi L, Tiseo D, Creti R, Baldassarri L, Pasanisi F, Contaldo F. "Central venous catheter infections and antibiotic therapy during long-term home parenteral nutrition: an 11-year follow-up study." *JPEN J Parenter Enteral Nutr. 2010 May-Jun;34(3):254-62. doi: 10.1177/0148607110362900.*

22. Bisseling TM, Willems MC, Versleijen MW, Hendriks JC, Vissers RK, Wanten GJ. "Taurolidine lock is highly effective in preventing catheter-related bloodstream infections in patients on home parenteral nutrition: a heparin-controlled prospective trial." *Clin Nutr. 2010 Aug;29(4):464-8. doi: 10.1016/j.clnu.2009.12.005. Epub 2010 Jan 12.*

23. Crispin A, Thul P, Arnold D, Schild S, Weimann A. "Central venous catheter complications during home parenteral nutrition: a prospective pilot study of 481 patients with more than 30,000 catheter days." *Onkologie. 2008 Nov;31(11):605-9. doi: 10.1159/000162286. Epub 2008 Oct 23.*

24. Clare A, Teubner A, Shaffer JL. "What information should lead to a suspicion of catheter sepsis in HPN?" *Clin Nutr. 2008 Aug;27(4):552-6. doi: 10.1016/j.clnu.2008.04.013. Epub 2008 Jul 26.*

25. Bonifacio R, Alfonsi L, Santarpia L, Orban A, Celona A, Negro G, Pasanisi F, Contaldo F. "Clinical outcome of long-term home parenteral nutrition in non-oncological patients: a report from two specialised centres." *Intern Emerg Med. 2007 Oct;2(3):188-95. Epub 2007 Oct 3.*

26. Ugur A, Marashdeh BH, Gottschalck I, Brøbech Mortensen P, Staun M, Bekker Jeppesen P. "Home parenteral nutrition in Denmark in the period from 1996 to 2001". *Scand J Gastroenterol. 2006 Apr;41(4):401-7.*

27. Shirotani N, Iino T, Numata K, Kameoka S. "Complications of central venous catheters in patients on home parenteral nutrition: an analysis of 68 patients over 16 years". *Surg Today. 2006;36(5):420-4.*

28. Chang A, Enns R, Saqui O, Chatur N, Whittaker S, Allard JP. "Line sepsis in home parenteral nutrition patients: are there socioeconomic risk factors? A Canadian study”. *JPEN J Parenter Enteral Nutr. 2005 Nov-Dec;29(6):408-12.*

29. Freshwater DA, Saadeddin A, Deel-Smith P, Digger T, Jones BJ. "Can home parenteral nutrition be provided by non-specialised centres? 2300 weeks of experience at a district general hospital in the United Kingdom”. *Clin Nutr. 2005 Apr;24(2):229-35.*

30 Pironi L, Paganelli F, Labate AM, Merli C, Guidetti C, Spinucci G, Miglioli M. "Safety and efficacy of home parenteral nutrition for chronic intestinal failure: a 16-year experience at a single centre”. *Dig Liver Dis. 2003 May;35(5):314-24.*

31. Bozzetti F, Mariani L, Bertinet DB, Chiavenna G, Crose N, De Cicco M, Gigli G, Micklewright A, Moreno Villares JM, Orban A, Pertkiewicz M, Pironi L, Vilas MP, Prins F, Thul P. "Central venous catheter complications in 447 patients on home parenteral nutrition: an analysis of over 100.000 catheter days”. *Clin Nutr. 2002 Dec;21(6):475-85.*

32. Santarpia L, Pasanisi F, Alfonsi L, Violante G, Tiseo D, De Simone G, Contaldo F. "Prevention and treatment of implanted central venous catheter (CVC) - related sepsis: a report after six years of home parenteral nutrition (HPN)." *Clin Nutr. 2002 Jun;21(3):207-11.*

33. Reimund JM, Arondel Y, Finck G, Zimmermann F, Duclos B, Baumann R. "Catheter-related infection in patients on home parenteral nutrition: results of a prospective survey." *Clin Nutr. 2002 Feb;21(1):33-8.*

34. Guedon C, Nouvellon M, Lalaude O, Lerebours E. "Efficacy of antibiotic-lock technique with teicoplanin in staphylococcus epidermidis catheter-related sepsis during long-term parenteral nutrition." *JPEN J Parenter Enteral Nutr. 2002 Mar-Apr;26(2):109-13.*

35. Terra RM, Plopper C, Waitzberg DL, Cukier C, Santoro S, Martins JR, Song RJ, Gama-Rodrigues J. "Remaining small bowel length: association with catheter sepsis in patients receiving home total parenteral nutrition: evidence of bacterial translocation." *World J Surg. 2000 Dec;24(12):1537-41.*

36. Bier ID. "Peripheral intravenous nutrition therapy: outpatient, office-based administration." Altern Med Rev. 2000 Aug; 5(4): 347-54.

37. Reimund J, Duclos B, Cuby C, Malzac D, Zimmermann F, Dietemann JL, Beretz L, Baumann R. "Home parenteral nutrition: clinical and laboratory analysis of initial experience (1994-1997). Implications for patient management." *Ann Nutr Metab. 1999; 43(6):329-38.*

38. Shields PL, Field J, Rawlings J, Kendall J, Allison SP. "Long-term outcome and cost-effectiveness of parenteral nutrition for acute gastrointestinal failure" *Clin Nutr. 1996 Apr;15(2):64-8.*

39. Allen ED, Mick AB, Nicol J, McCoy KS. Prolonged parenteral nutrition for cystic fibrosis patients. *Nutr Clin Pract. 1995 Apr;10(2):73-9.*

40. Williams N, Scott NA, Irving MH. Catheter-related morbidity in patients on home parenteral nutrition: implications for small bowel transplantation. *Ann R Coll Surg Engl. 1994 Nov;76(6):384-6.*

41. Buchman AL, Moukarzel A, Goodson B, Herzog F, Pollack P, Reyen L, Alvarez M, Ament ME, Gornbein J. Catheter-related infections associated with home parenteral nutrition and predictive factors for the need for catheter removal in their treatment. *JPEN J Parenter Enteral Nutr. 1994 Jul-Aug;18(4):297-302. Erratum in: JPEN J Parenter Enteral Nutr 1997 Mar-Apr;21(2):117.*

42. O'Keefe SJ, Burnes JU, Thompson RL. Recurrent sepsis in home parenteral nutrition patients: an analysis of risk factors. *JPEN J Parenter Enteral Nutr. 1994 May-Jun;18(3):256-63*

43. Williams N, Carlson GL, Scott NA, Irving MH. Incidence and management of catheter-related sepsis in patients receiving home parenteral nutrition. *Br J Surg. 1994 Mar;81(3):392-4.*

44. Pironi L, Miglioli M, Ruggeri E, Longo N, Suriani U, Maselli S, Gnudi S, Barbara L. Home parenteral nutrition for the management of chronic intestinal failure: a 34 patient-year experience. *Ital J Gastroenterol. 1993 Oct;25(8):411-8.*

45. Nance ML, Morris JB, Mullen JL. Home parenteral nutrition after near total enterectomy. *J Am Coll Nutr. 1993 Jun;12(3):281-5.*

46. Burnes JU, O'Keefe SJ, Fleming CR, Devine RM, Berkner S, Herrick L. Home parenteral nutrition--a 3-year analysis of clinical and laboratory monitoring. *JPEN J Parenter Enteral Nutr. 1992 Jul-Aug;16(4):327-32.*

47. Herfindal ET, Bernstein LR, Wong AF, Hogue VW, Darbinian JA. Complications of home parenteral nutrition. *Clin Pharm. 1992 Jun;11(6):543-8.*

48. Singer P, Rothkopf MM, Kvetan V, Kirvelä O, Gaare J, Askanazi J. Risks and benefits of home parenteral nutrition in the acquired immunodeficiency syndrome. *JPEN J Parenter Enteral Nutr. 1991 Jan-Feb;15(1):75-9.*

49. Galandiuk S, O'Neill M, McDonald P, Fazio VW, Steiger E. A century of home parenteral nutrition for Crohn's disease. *Am J Surg. 1990 Jun;159(6):540-4; discussion 544-5.*

50. Herfindal ET, Bernstein LR, Kudzia K, Wong A. Survey of home nutritional support patients. *JPEN J Parenter Enteral Nutr. 1989 May-Jun;13(3):255-61.*

51. Messing B, Landais P, Goldfarb B, Irving M. Home parenteral nutrition in adults: a multicentre survey in Europe. *Clin Nutr. 1989 Feb;8(1):3-9.*

52. Messing B, Peitra-Cohen S, Debure A, Beliah M, Bernier JJ. Antibiotic-lock technique: a new approach to optimal therapy for catheter-related sepsis in home-parenteral nutrition patients. *JPEN J Parenter Enteral Nutr. 1988 Mar-Apr;12(2):185-9.*

53. Gouttebel MC, Saint-Aubert B, Jonquet O, Astre C, Joyeux H. Ambulatory home total parenteral nutrition. *JPEN J Parenter Enteral Nutr. 1987 Sep-Oct;11(5):475-9.*

54. Mughal M, Irving M. Home parenteral nutrition in the United Kingdom and Ireland. *Lancet. 1986 Aug 16;2(8503):383-7.*

55. Wolfe BM, Ryder MA, Nishikawa RA, Halsted CH, Schmidt BF. Complications of parenteral nutrition. *Am J Surg. 1986 Jul;152(1):93-9.*

56. Rannem T, Ladefoged K, Tvede M, Lorentzen JE, Jarnum S. Catheter-related septicaemia in patients receiving home parenteral nutrition. *Scand J Gastroenterol. 1986 May;21(4):455-60.*

57. Gaggioti G, Orlandoni P, Boccoli G, Capomagi A, Talevi S, Ambrosi S. Percutaneous vs. totally implantable catheters in home parenteral nutrition. *Clin Nutr. 1986 Feb;5(1):33-40.*

58. Dudrick SJ, O'Donnell JJ, Englert DM, Matheny RG, Blume ER, Nutt RE, Hickey MS, Barroso AO. 100 patient-years of ambulatory home total parenteral nutrition. *Ann Surg. 1984 Jun;199(6):770-81.*

59. Slocombe GW, Ham R, Lord G, Newland AC. Long-term home parenteral nutrition. *Br J Hosp Med. 1983 Jul;30(1):82.*

60. McCannon GD. Aspects of IV therapy. 2. Parenteral nutrition in the USA. *Nurs Times. 1983 May 11-17;79(19):28, 30-3.*

61. Lees CD, Steiger E, Hooley RA, Montague N, Srp F, Gulledge AD, Wateska LP, Frame C. Home parenteral nutrition. *Surg Clin North Am. 1981 Jun;61(3):621-33.*

62. Pollack PF, Kadden M, Byrne WJ, Fonkalsrud EW, Ament ME. 100 patient years' experience with the Broviac silastic catheter for central venous nutrition. *JPEN J Parenter Enteral Nutr. 1981 Jan-Feb;5(1):32-6.*

63. Fleming CR, Witzke DJ, Beart RW Jr. Catheter-related complications in patients receiving home parenteral nutrition. *Ann Surg. 1980 Nov;192(5):593-9.*

64. Miller DG, Ivey M, Ivey T, Scribner BH. Experience with an indwelling right atrial catheter for home parenteral nutrition. *Surg Gynecol Obstet. 1980 Jul;151(1):108-10.*

65. Ladefoged K, Jarnum S. Long-term parenteral nutrition. *Br Med J. 1978 Jul 22;2(6132):262-6.*

66. Jeejeebhoy KN, Langer B, Tsallas G, Chu RC, Kuksis A, Anderson GH. Total parenteral nutrition at home: studies in patients surviving 4 months to 5 years. *Gastroenterology. 1976 Dec;71(6):943-53.*

67. Riella MC, Scribner BH. Five years' experience with a right atrial catheter for prolonged parenteral nutrition at home. *Surg Gynecol Obstet. 1976 Aug;143(2):205-8.*

c. No infection rates per catheter reported.

1. Bond, A., A. Teubner, M. Taylor, C. Cawley, J. Varden, A. Abraham, P. R. Chadwick, M. Soop, G. L. Carlson, and S. Lal. "Catheter-Related Infections in Patients with Acute Type Ii Intestinal Failure Admitted to a National Centre: Incidence and Outcomes." *Clin Nutr* 38 no. 4 (2019): 1828-32.

2. Emery, D., A. Pearson, R. Lopez, C. Hamilton, and N. M. Albert. "Voiceover Interactive Powerpoint Catheter Care Education for Home Parenteral Nutrition." *Nutr Clin Pract* 30, no. 5 (2015): 714-9.

3. Gomez-Candela, C., M. Martin Fuentes, N. Garcia Vazquez, M. Crespo Yanguas, A. Lisbona Catalan, R. Campos Del Portillo, and S. Palma Milla. "Twenty-Five Years of Home Parenteral Nutrition Outsourcing: The Experience at Hospital Universitario La Paz, Madrid." *Nutr Hosp* 30, no. 6 (2014): 1295-302.

4. Higuera, I., P. Garcia-Peris, M. Camblor, I. Breton, C. Velasco, R. Romero, L. Frias, and C. Cuerda. "Outcomes of a General Hospital-Based Home Parenteral Nutrition (Hpn) Program; Report of Our Experience from a 26-Year Period." *Nutr Hosp* 30, no. 2 (2014): 359-65.

5. Hortencio, T. D. R., B. M. Arendt, A. Teterina, K. N. Jeejeebhoy, L. M. Gramlich, J. S. Whittaker, D. Armstrong, M. Raman, R. J. N. Nogueira, and J. P. Allard. "Changes in Home Parenteral Nutrition Practice Based on the Canadian Home Parenteral Nutrition Patient Registry." *JPEN J Parenter Enteral Nutr* 41, no. 5 (2017): 830-36.

6. Obling, S. R., B. V. Wilson, and J. Kjeldsen. "Home Parenteral Support in Patients with Incurable Cancer. Patient Characteristics of Importance for Catheter Related Complications and Overall Survival." *Clin Nutr ESPEN* 28 (2018): 88-95.

7. Santarpia, L., A. Buonomo, M. C. Pagano, L. Alfonsi, M. Foggia, M. Mottola, G. Z. Marinosci, F. Contaldo, and F. Pasanisi. "Central Venous Catheter Related Bloodstream Infections in Adult Patients on Home Parenteral Nutrition: Prevalence, Predictive Factors, Therapeutic Outcome." *Clin Nutr* 35, no. 6 (2016): 1394-98.

d. Other missing data for extraction.

1. Allan, P. J., P. Stevens, A. Abraham, P. Paine, K. Farrer, A. Teubner, G. Carlson, and S. Lal. "Outcome of Intestinal Failure after Bariatric Surgery: Experience from a National Uk Referral Centre." *Eur J Clin Nutr* 70, no. 7 (2016): 772-8.

2. Bell, A., N. Conway, J. Courtney, K. Kennedy, Z. Raubenheimer, N. Rice, D. Kevans, C. L. Donohoe, and J. V. Reynolds. "Point Prevalence of Adult Intestinal Failure in Republic of Ireland." *Ir Med J* 111, no. 2 (2018): 688.

3. Brandt, C. F., S. Tribler, M. Hvistendahl, R. M. Naimi, P. Brobech, M. Staun, and P. B. Jeppesen. "Home Parenteral Nutrition in Adult Patients with Chronic Intestinal Failure: Catheter-Related Complications over 4 Decades at the Main Danish Tertiary Referral Center." *JPEN J Parenter Enteral Nutr* 42, no. 1 (2018): 95-103.

4. Corrigan, M. L., C. Pogatschnik, D. Konrad, and D. F. Kirby. "Hospital Readmissions for Catheter-Related Bloodstream Infection and Use of Ethanol Lock Therapy: Comparison of Patients Receiving Parenteral Nutrition or Intravenous Fluids in the Home Vs a Skilled Nursing Facility." *JPEN J Parenter Enteral Nutr* 37, no. 1 (2013): 81-4.

5. Cotogni, P., C. Barbero, C. Garrino, C. Degiorgis, B. Mussa, A. De Francesco, and M. Pittiruti. "Peripherally Inserted Central Catheters in Non-Hospitalized Cancer Patients: 5-Year Results of a Prospective Study." *Support Care Cancer* 23, no. 2 (2015): 403-9.

6. Dibb, M., M. Soop, A. Teubner, J. Shaffer, A. Abraham, G. Carlson, and S. Lal. "Survival and Nutritional Dependence on Home Parenteral Nutrition: Three Decades of Experience from a Single Referral Centre." *Clin Nutr* 36, no. 2 (2017): 570-76.

7. Huard, G., M. Bouin, M. Lemoyne, and L. D'Aoust. "Vertebral Osteomyelitis: An under-Recognized Infectious Complication in Patients on Home Parenteral Nutrition." *J Clin Med Res* 6, no. 4 (2014): 272-7.
8. Ross, V. M., P. Guenter, M. L. Corrigan, D. Kovacevich, M. F. Winkler, H. E. Resnick, T. L. Norris, L. Robinson, and E. Steiger. "Central Venous Catheter Infections in Home Parenteral Nutrition Patients: Outcomes from Sustain: American Society for Parenteral and Enteral Nutrition's National Patient Registry for Nutrition Care." *Am J Infect Control* 44, no. 12 (2016): 1462-68.
9. Szeinbach, S. L., J. Pauline, K. F. Villa, S. R. Commerford, A. Collins, and E. Seoane-Vazquez. "Evaluating Catheter Complications and Outcomes in Patients Receiving Home Parenteral Nutrition." *J Eval Clin Pract* 21, no. 1 (2015): 153-9.
10. Theilla, M., M. Lawinski, J. Cohen, E. Hadar, I. Kagan, M. Perkewick, and P. Singer. "Safety of Home Parenteral Nutrition During Pregnancy." *Clin Nutr* 36, no. 1 (2017): 288-92.
11. Uzzan, M., J. Kirchgesner, J. Poupon, O. Corcos, I. Pingenot, and F. Joly. "Antioxidant Trace Elements Serum Levels in Long-Term Parenteral Nutrition (Pn): Prevalence and Infectious Risk Associated with Deficiencies, a Retrospective Study from a Tertiary Home-Pn Center." *Clin Nutr* 36, no. 3 (2017): 812-17.
12. Vallabh, H., D. Konrad, R. DeChicco, G. Cresci, R. Lopez, E. Steiger, and D. F. Kirby. "Thirty-Day Readmission Rate Is High for Hospitalized Patients Discharged with Home Parenteral Nutrition or Intravenous Fluids." *JPEN J Parenter Enteral Nutr* 41, no. 8 (2017): 1278-85.
13. Van Gossum, A., L. Pironi, C. Chambrier, M. Dreesen, C. F. Brandt, L. Santarpia, and F. Joly. "Home Parenteral Nutrition (Hpn) in Patients with Post-Bariatric Surgery Complications." *Clin Nutr* 36, no. 5 (2017): 1345-48.
14. Wu, G., Y. Jiang, X. Zhu, D. Jin, Y. Han, J. Han, Z. Wu, and Z. Wu. "Prevalence and Risk Factors for Complications in Adult Patients with Short Bowel Syndrome Receiving Long-Term Home Parenteral Nutrition." *Asia Pac J Clin Nutr* 26, no. 4 (2017): 591-97.
15. John BK, Khan MA, Speerhas R, Rhoda K, Hamilton C, Dechicco R, Lopez R, Steiger E, Kirby DF. "Ethanol lock therapy in reducing catheter-related bloodstream infections in adult home parenteral nutrition patients: results of a retrospective study." *JPEN J Parenter Enteral Nutr. 2012 Sep;36(5):603-10. doi: 10.1177/0148607111428452. Epub 2011 Dec 28.*

16. Gillanders L, Angstmann K, Ball P, O'Callaghan M, Thomson A, Wong T, Thomas M. "A prospective study of catheter-related complications in HPN patients." *Clin Nutr. 2012 Feb;31(1):30-4. doi: 10.1016/j.clnu.2011.09.009. Epub 2011 Oct 4.*

17. Huisman-de Waal G, Versleijen M, van Achterberg T, Jansen JB, Sauerwein H, Schoonhoven L, Wanten G. "Psychosocial complaints are associated with venous access-device related complications in patients on home parenteral nutrition." *JPEN J Parenter Enteral Nutr. 2011 Sep;35(5):588-95. doi: 10.1177/0148607110385818.*

18. Brown M, Teubner A, Shaffer J, Herrick AL. "Home parenteral nutrition--an effective and safe long-term therapy for systemic sclerosis-relatedintestinal failure." Rheumatology (Oxford). 2008 Feb;47(2):176-9. Epub 2007 Dec 14.

19. Wang MY, Wu MH, Hsieh DY, Lin LJ, Lee PH, Chen WJ, Lin MT. "Home parenteral nutrition support in adults: experience of a medical center in Asia." *JPEN J Parenter Enteral Nutr. 2007 Jul-Aug;31(4):306-10.*

20. Opilla MT, Kirby DF, Edmond MB. "Use of ethanol lock therapy to reduce the incidence of catheter-related bloodstream infections in home parenteral nutrition patients." *JPEN J Parenter Enteral Nutr. 2007 Jul-Aug;31(4):302-5.*

21. Marra AR, Opilla M, Edmond MB, Kirby DF. "Epidemiology of bloodstream infections in patients receiving long-term total parenteral nutrition." J *Clin Gastroenterol. 2007 Jan;41(1):19-28.*

22. Violante G, Alfonsi L, Santarpia L, Cillis MC, Negro G, De Caprio C, Russo N, Contaldo F, Pasanisi F. "Adult home parenteral nutrition: a clinical evaluation after a 3-year experience in a Southern European centre." *Eur J Clin Nutr. 2006 Jan;60(1):58-61.*

23. Jurewitsch B, Jeejeebhoy KN. "Taurolidine lock: the key to prevention of recurrent catheter-related bloodstream infections." *Clin Nutr. 2005 Jun;24(3):462-5. Epub 2005 Apr 22.*

24. Ireton-Jones C, DeLegge M. "Home parenteral nutrition registry: a five-year retrospective evaluation of outcomes of patients receiving home parenteral nutrition support." *Nutrition. 2005 Feb;21(2):156-60.*

25. Van Gossum A, Vahedi K, Abdel-Malik, Staun M, Pertkiewicz M, Shaffer J, Hebuterne X, Beau P, Guedon C, Schmit A, Tjellesen L, Messing B, Forbes A; ESPEN-HAN Working Group. "Clinical,

social and rehabilitation status of long-term home parenteral nutrition patients: results of a European multicentre survey." *Clin Nutr. 2001 Jun;20(3):205-10.*

e. Duplicated published series

1. Bech, L. F., L. Drustrup, L. Nygaard, A. Skallerup, L. D. Christensen, L. Vinter-Jensen, H. H. Rasmussen, and M. Holst. "Environmental Risk Factors for Developing Catheter-Related Bloodstream Infection in Home Parenteral Nutrition Patients: A 6-Year Follow-up Study." *JPEN J Parenter Enteral Nutr* 40, no. 7 (2016): 989-94.
2. Botella-Carretero, J. I., C. Carrero, E. Guerra, B. Valbuena, F. Arrieta, A. Calanas, I. Zamarron, J. A. Balsa, and C. Vazquez. "Role of Peripherally Inserted Central Catheters in Home Parenteral Nutrition: A 5-Year Prospective Study." *JPEN J Parenter Enteral Nutr* 37, no. 4 (2013): 544-9.

f. Other reasons (including several of the above reasons and others such as different types of reported outcomes apart from catheter-related infections, use of locks as preventive measures, or type of lipid emulsions and other unrelated outcomes)

1. Al-Amin, A. H., J. Sarveswaran, J. M. Wood, D. A. Burke, and C. F. Donnellan. "Efficacy of Taurolidine on the Prevention of Catheter-Related Bloodstream Infections in Patients on Home Parenteral Nutrition." *J Vasc Access* 14, no. 4 (2013): 379-82.
2. Aria Guerra, E., A. Cortes-Salgado, R. Mateo-Lobo, L. Nattero, J. Riveiro, B. Vega-Pinero, B. Valbuena, F. Carabana, C. Carrero, E. Grande, A. Carrato, and J. I. Botella-Carretero. "Role of Parenteral Nutrition in Oncologic Patients with Intestinal Occlusion and Peritoneal Carcinomatosis." *Nutr Hosp* 32, no. 3 (2015): 1222-7.
3. Arnoriaga Rodriguez, M., M. Perez de Ciriza Cordeu, M. Camblor Alvarez, I. Breton Lesmes, M. Motilla de la Camara, C. Velasco Gimeno, L. Arhip, P. Garcia Peris, and C. Cuerda Compes. "Clinical and Economic Impact of the Taurolidine Lock on Home Parenteral Nutrition." *Nutr Hosp* 35, no. 4 (2018): 761-66.
4. Bond, A., D. H. Vasant, W. Gashau, A. Abraham, A. Teubner, K. Farrer, G. Leahy, and S. Lal. "Managing Successful Pregnancies in Patients with Chronic Intestinal Failure on Home Parenteral

Nutrition: Experience from a Uk National Intestinal Failure Unit." *J Gastrointestin Liver Dis* 26, no. 4 (2017): 375-79.

5. Christensen, L. D., H. H. Rasmussen, and L. Vinter-Jensen. "Peripherally Inserted Central Catheter for Use in Home Parenteral Nutrition: A 4-Year Follow-up Study." *JPEN J Parenter Enteral Nutr* 38, no. 8 (2014): 1003-6.
6. Davidson, J. B., J. Edakkanambeth Varayil, A. Okano, J. A. Whitaker, S. L. Bonnes, D. G. Kelly, M. S. Mundi, and R. T. Hurt. "Prevention of Subsequent Catheter-Related Bloodstream Infection Using Catheter Locks in High-Risk Patients Receiving Home Parenteral Nutrition." *JPEN J Parenter Enteral Nutr* 41, no. 4 (2017): 685-90.
7. Magambo, W. "Catheter-Related Infections in Patients on Home Parenteral Nutrition." *Br J Community Nurs* Suppl Nutrition (2013): S14-9.
8. Mundi, M. S., M. T. McMahon, J. J. Carnell, and R. T. Hurt. "Long-Term Use of Mixed-Oil Lipid Emulsion in Adult Home Parenteral Nutrition Patients: A Case Series." *Nutr Clin Pract* 33, no. 6 (2018): 851-57.
9. Saunders, J., M. Naghibi, Z. Leach, C. Parsons, A. King, T. Smith, and M. Stroud. "Taurolidine Locks Significantly Reduce the Incidence of Catheter-Related Blood Stream Infections in High-Risk Patients on Home Parenteral Nutrition." *Eur J Clin Nutr* 69, no. 2 (2015): 282-4.
10. Stanga, Z., C. Aeberhard, P. Scharer, A. Kocher, S. Adler, and P. M. Villiger. "Home Parenteral Nutrition Is Beneficial in Systemic Sclerosis Patients with Gastrointestinal Dysfunction." *Scand J Rheumatol* 45, no. 1 (2016): 32-35.
11. Tamiya, H., H. Yasunaga, H. Matusi, K. Fushimi, M. Akishita, and S. Ogawa. "Comparison of Short-Term Mortality and Morbidity between Parenteral and Enteral Nutrition for Adults without Cancer: A Propensity-Matched Analysis Using a National Inpatient Database." *Am J Clin Nutr* 102, no. 5 (2015): 1222-8.
12. Vasant, D. H., R. Kalaiselvan, J. Ablett, A. Bond, A. Abraham, A. Teubner, D. Green, P. A. Paine, and S. Lal. "The Chronic Intestinal Pseudo-Obstruction Subtype Has Prognostic Significance in Patients with Severe Gastrointestinal Dysmotility Related Intestinal Failure." *Clin Nutr* 37, no. 6 Pt A (2018): 1967-75.

13. Wouters, Y., B. Roosenboom, E. Causevic, W. Kievit, H. Groenewoud, and G. J. A. Wanten. "Clinical Outcomes of Home Parenteral Nutrition Patients Using Taurolidine as Catheter Lock: A Long-Term Cohort Study." *Clin Nutr* (2018).
14. Wouters, Y., M. Theilla, P. Singer, S. Tribler, P. B. Jeppesen, L. Pironi, L. Vinter-Jensen, H. H. Rasmussen, F. Rahman, and G. J. A. Wanten. "Randomised Clinical Trial: 2% Taurolidine Versus 0.9% Saline Locking in Patients on Home Parenteral Nutrition." *Aliment Pharmacol Ther* 48, no. 4 (2018): 410-22.
15. Wouters, Y., R. K. Vissers, H. Groenewoud, W. Kievit, and G. J. A. Wanten. "Repair of Damaged Central Venous Catheters Is Safe and Doubles Catheter Survival: A Home Parenteral Nutrition Patient Cohort Study." *Clin Nutr* (2018).
16. Pironi L, Joly F, Forbes A, Colomb V, Lyszkowska M, Baxter J, Gabe S, Hébuterne X, Gambarara M, Gottrand F, Cuerda C, Thul P, Messing B, Goulet O, Staun M, Van Gossum A; Home Artificial Nutrition & Chronic Intestinal Failure Working Group of the European Society for Clinical Nutritionand Metabolism (ESPEN). "Long-term follow-up of patients on home parenteral nutrition in Europe: implications for intestinal transplantation." *Gut. 2011 Jan;60(1):17-25. doi: 10.1136/gut.2010.223255. Epub 2010 Nov 10.*
17. Smith CE, Curtas S, Werkowitch M, Kleinbeck SV, Howard L. "Home parenteral nutrition: does affiliation with a national support and educational organization improve patient outcomes?" *JPEN J Parenter Enteral Nutr*. 2002 May-Jun;26(3):159-63.
18. Van Gossum A. "Clinical profile of home parenteral nutrition patients" *Acta Gastroenterol Belg.* 1995 Sep-Dec;58(5-6):366-9.
19. Messing B. "Catheter-related sepsis during home parenteral nutrition" *Clin Nutr.* 1995 Jun;14 Suppl 1:46-51.
20. Steiger E. "Home parenteral nutrition. Components, application, and complications" *Postgrad Med.* 1984 May;75(6):95-102.
21. Muller RJ, Hoffman DM, Mulligan RM. "Parenteral nutrition program in a major cancer center" *Hosp Pharm.* 1981 Feb;16(2):54-66.
22. Byrne WJ, Ament ME, Burke M, Fonkalsrud E. "Home parenteral nutrition". *Surg Gynecol Obstet.* 1979 Oct;149(4):593-99.
